# Optimization of MOEMS Projection Module Performance with Enhanced Piezoresistive Sensitivity

**DOI:** 10.3390/mi11070651

**Published:** 2020-06-30

**Authors:** Huijun Yu, Peng Zhou, Kewei Wang, Yanfei Huang, Wenjiang Shen

**Affiliations:** 1 School of Nano-Tech and Nano-Bionics, University of Science and Technology of China, Hefei 230026, China; hjyu2012@sinano.ac.cn; 2 Key Lab of Nanodevices and Applications, Suzhou Institute of Nano-Tech and Nano-Bionics, Chinese Academy of Sciences, Suzhou 215123, China; pzhou2015@sinano.ac.cn (P.Z.); kwwang2012@sinano.ac.cn (K.W.); yfhuang2016@sinano.ac.cn (Y.H.)

**Keywords:** MOEMS micromirror, sensitivity of piezoresistive sensor, zero-crossing time, position estimation, error of the image pixel

## Abstract

In scanning laser projection systems, the laser modulation time is important for the projection resolution. The modulation time needs to be matched with the motion of the micromirror. For this paper, the piezoresistive sensor was integrated on the torsion beam of the micromirror to monitor the physical position of the micromirror. The feedback signal was used to generate the zero-crossing time, which was used to estimate the physical position of the resonating mirror over time. The estimated position was affected by the zero-crossing time and the error directly influenced the definition of the projected image. By reducing the impurity concentration from 3 × 10^18^/cm^3^ to 1 × 10^18^/cm^3^ and increasing shear stress on piezoresistive sensor, the sensitivity of the piezoresistive sensor increased from 4.4 mV/V° to 6.4 mV/V° and the error of the image pixel reduced from 1.5 pixels to 0.5 pixels. We demonstrated that the image quality of an Optical-Microeletromechanical Systems (MOEMS) laser projection could be improved by enhancing the sensitivity of the piezoresistive sensor.

## 1. Introduction

The benefits of miniaturization through Micro-opto-electro-mechanical Systems (MOEMS) can be seen in many applications, especially in optics and photonics. Micromirror is a kind of optical—Microelectromechanical systems (MEMS) device that has a vast array of applications due to its high performance, low production cost, small size, and low power consumption [1,2,3]. It has been widely used in laser projection, barcode scanners, confocal microscopy, finger printing sensing, head-up displays, optical coherence tomography (OCT) and Lidar [4,5,6,7,8,9,10]. For the application of laser projection, the displays are created by using a micromirror to scan a modulated light beam to cover the desired field of view [11,12,13,14,15,16,17]. The micromirror can be a single dual-axis mirror or two separate, orthogonal single-axis mirrors that include a fast scan mirror and slow scan mirror. The light consists of three laser colors, red, green and blue (RGB). RGB lasers need to be modulated simultaneously, in order to create the proper color mix for each pixel [18].

A scanning laser projector is an example of a resonant system. It needs a mirror which is resonant in at least one dimension. In order to keep the micromirror at a resonance frequency and phase, it is necessary to have a feedback signal to conduct closed-loop control [19,20,21,22]. The common feedback methods include optical, electrostatic, magnetic and piezoresistive. For mass production and miniaturization, a piezoresistive sensor is used as a feedback element. The piezoresistive sensor is integrated on the torsional beam of the micromirror to generate feedback signals. Scanning laser projectors use parameter estimation circuits which include filters, comparators to estimate the operating parameters such as phase shift, period and zero-crossing time. The zero-crossing time is used to estimate the physical position of the resonating mirror over time. The image resolution of scanning laser projector is limited by the accuracy with which the position of the resonating mirror can be estimated. This paper presents the relationship between the feedback signal and the position estimation accuracy. The enhancement of the feedback signal improves the signal-to-noise ratio (SNR) of the comparator signal, so as to improve the accuracy of the zero-crossing time judgment. It increases the position estimation accuracy and reduces the influence of the circuit noise on the quality of projection imaging. As the position estimation accuracy increases, pixels can be placed more precisely during the oscillation of the mirror, thereby improving image quality.

Section 2 describes the design of piezoresistive sensor. The sensitivity of piezoresistive sensor is improved by optimizing design of the piezoresistive sensor. Section 3 gives the fabrication process of the micromirror with integrated piezoresistive sensor. Section 4 presents the measurement results about the optimized piezoresistive sensor of the micromirror. In Section 5, the influence of the feedback signal on the error of the image pixel in scanning laser projection is discussed. Finally, Section 6 concludes the main remarks for this paper.

## 2. Principle and Design of Piezoresistive Sensor

**Figure 1:**
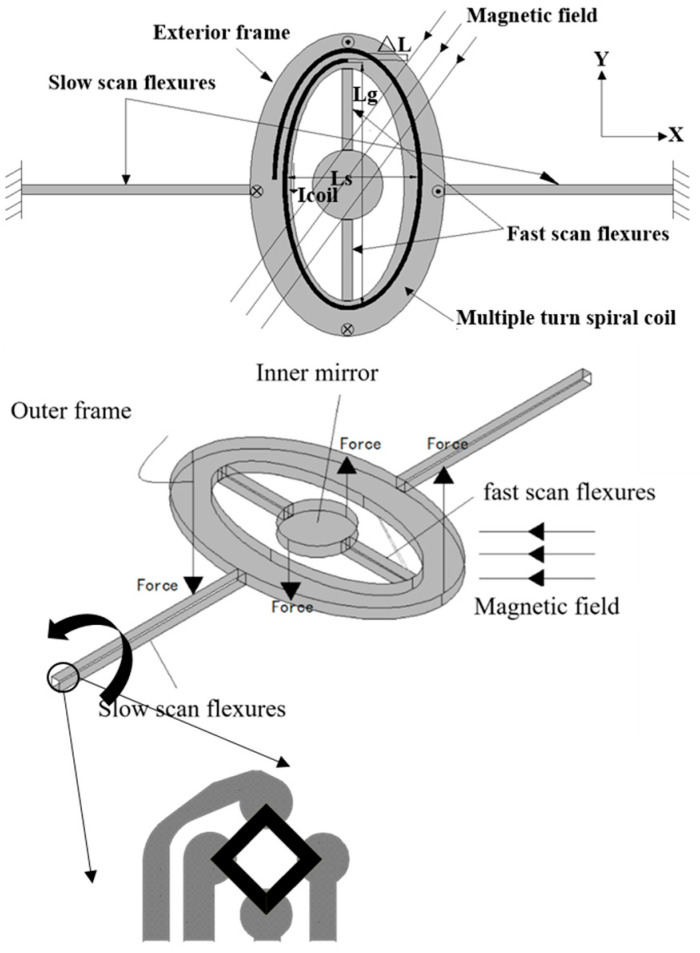
The basic structure of microeletromechanical systems (MEMS) micromirror with integrated piezoresistive sensor.

As shown in Figure 1, the micromirror is connected to an exterior frame through the fast scan flexures. The inner mirror can rotate along the fast scan flexures to achieve the scan in x-direction. The outer frame connects to the substrate through the slow scan flexures so that the frame and mirror can rotate together along the slow scan flexures to achieve the scan in y-direction. A metal coil with multiple turns is electroplated on the surface of the exterior frame. A magnetic field is applied in the mirror surface and has 45° angles relative to both fast and slow scan flexures, where both components of magnetic field along x-axis and y-axis are applied. When the driving current flows through the metal coil, the Lorentz force, which is out of the plane, is generated and acts on the current carrying metal coil. The driving current is a mixed signal composed of two sinusoidal signals, a low frequency signal and a high frequency signal. Since the metal coil forms loops on the exterior frame, the equivalent Lorentz forces form two pairs of torques along the scan flexures in both directions, which can drive the micromirror to scan in both directions depending on the frequency of the driving signals [23]. The piezoresistive sensor is a Wheatstone bridge with four piezoresistive bars which is placed on the torsional beam of the micromirror, as shown in Figure 1. The shear stress makes the piezoresistive element distort and changes the resistivity of the piezoresistive element. When the mirror is working, the scan flexures deform. The shear stress caused by the deformation of the scan flexures results in the output of the piezoresistive sensor. The shear stress of the torsional beam is proportional to the rotation angle of the mirror [24]. The output of the piezoresistive sensor is a voltage signal. This section focuses on improving the voltage signal at a fixed supply voltage of the piezoresistive sensor.

**Figure 2:**
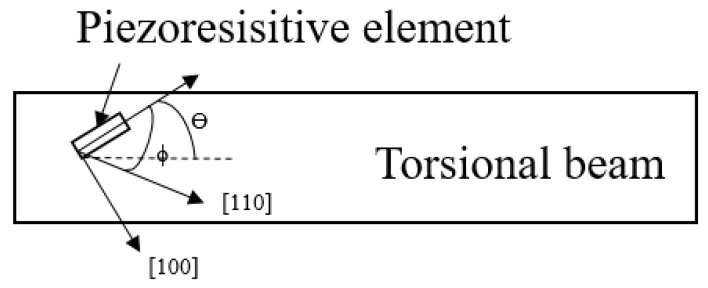
Torsional beam and piezoresistive element on silicon(100) wafer.

First, it is necessary to determine the doping type of the piezoresistive sensor and the direction of the torsional beam. Figure 2 shows the piezoresistive element in the torsional beam. It forms an angle *θ* with the direction of the torsional beam and *ϕ* with the <110> direction. As the micromirror is twisting, it has a considerable distribution of shear stress inside the torsion bar. The shear stress reached the maximum at the top surface when the cross section of the beam is a long vertical rectangle. So, Equation (1) presents the output voltage of the piezoresistive sensor which is only affected by shear stress [25].

(1) $$V_{\mathrm{output}}=\frac{W}{L}V\frac{\rho_{6}}{\rho_{1}}=\frac{W}{L}V\left[\sin\left(2\theta \right)\sin\left(2\phi \right)\left(\pi_{11}-\pi_{12}\right)+\cos\left(2\theta \right)\cos\left(2\phi \right)\pi_{44}\right]T_{6}$$

*V* is the supply voltage. *W* and *L* are the width and length of the piezoresistive sensor. *π*_11_, *π*_12_ and *π*_44_ are the piezoresistive coefficients. *T*_6_ is the shear stress. Through the calculation by Equation (1), the sensitivity of n-type under the action of shear stress is higher than that of p-type piezoresistive sensor when the torsional beam is along the <110> direction. The calculated results of the piezoresistive coefficients are shown in Table 1. When *θ* = *ϕ* = 45° and the torsion beam are along the <110> direction, it has a maximum piezoresistive coefficient.

The signal *V*_output_ is expressed as follows [26,27]:

(2) $$V_{\mathrm{output}}=\frac{W}{L}V\left(\pi_{11}-\pi_{12}\right)T_{6}$$

*V* is the supply voltage, and maintains a stable value of 3.3 V. From Equation (2), the signal *V*_output_ can be improved by increasing the supply voltage, piezoresistive coefficient and shear stress. The high supply voltage increases the heat and power dissipation of the piezoresistive sensor. And as the piezoresistive sensor is embedded in the torsion beam, the increasing of the heat will directly affect the performance of the torsion beam and the resonance frequency of the micromirror. So it is generally not possible to improve the signal *V*_output_ by increasing the supply voltage. When the supply voltage is fixed, the sensitivity of the piezoresistive sensor can be improved by optimizing the piezoresistive coefficient and increasing the shear stress.

The piezoresistive coefficient is directly related to the impurity concentration. Cho reported the experience equations of piezoresistive coefficient and impurity concentration(*C*) [28].

(3) $$\pi_{11}^{n}=71.695Ln\left(C\right)-3739.6,$$

(4) $$\pi_{12}^{n}=-23.479Ln\left(C\right)-1319.6.$$

**Figure 3:**
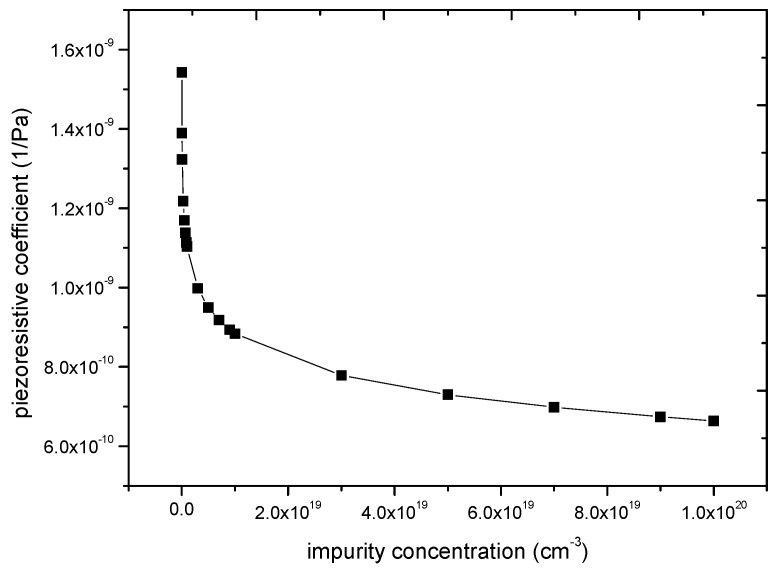
N-type piezoresistive coefficient (*π*_11_ − *π*_12_) of different impurity concentrations.

**Figure 4:**
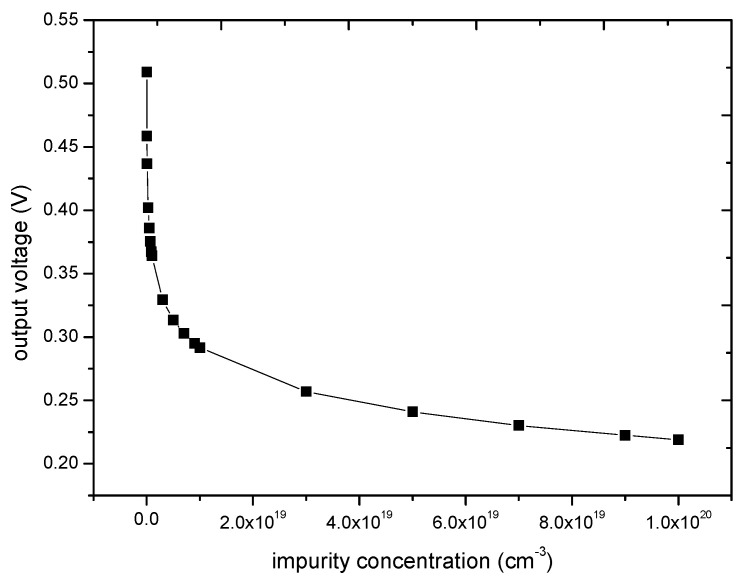
The relation between output voltage and doping concentration.

Through Equations (3) and (4), Figure 3 shows the value of piezoresistive coefficient (*π*_11_ − *π*_12_) in different impurity concentrations. Assuming that the shear stress is 100 MPa, the supply voltage is 3.3 V, the relation between piezoresistive coefficient and impurity concentration can be obtained by theoretical calculation. Figure 4 shows the relationship between the output voltage and impurity concentration. In this paper, a group of experiments are carried out to verify the feasibility of improving the piezoresistive sensitivity by reducing the impurity concentration. The impurity concentration of 1 × 10^18^/cm^3^ and 3 × 10^18^/cm^3^ is chosen. From theoretical calculations, the output voltage of the former is 11% higher than that of the latter, as shown in Figure 4.

**Figure 5:**
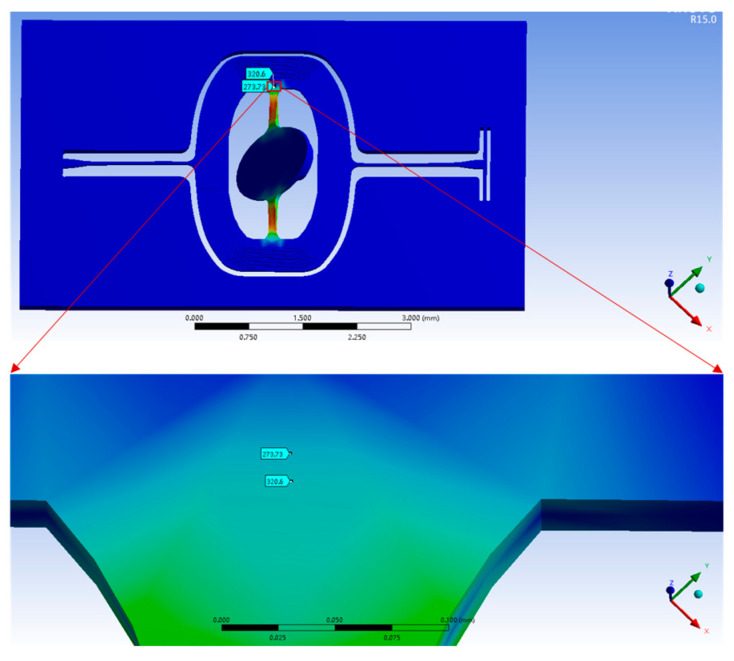
The shear stress at different positions on the torsional beam.

The piezoresistive sensor is placed at the end of the torsional beam of the micromirror. Without changing the size of the torsional beam, the piezoresistive sensor is placed where the shear stress is higher. This paper gives a set of comparisons at different positions of the torsional beam. Figure 5 shows the simulation results of the shear stress. In the designs of the micromirror, the piezoresistive sensor places in two positions, where the shear stresses are 273 MPa and 320 MPa, respectively. Due to the need of the working frequency of the micromirror, the size of the torsion beam of the micromirror cannot change. Piezoresistive sensors and corresponding wires cannot be placed at the position in which the shear stress is 1000 MPa due to size constraints. 320 MPa is the maximum stress that can be placed. Theoretically, the piezoresistive output is increasing by 17% when the other parameters are unchanged.

## 3. Fabrication

**Figure 6:**
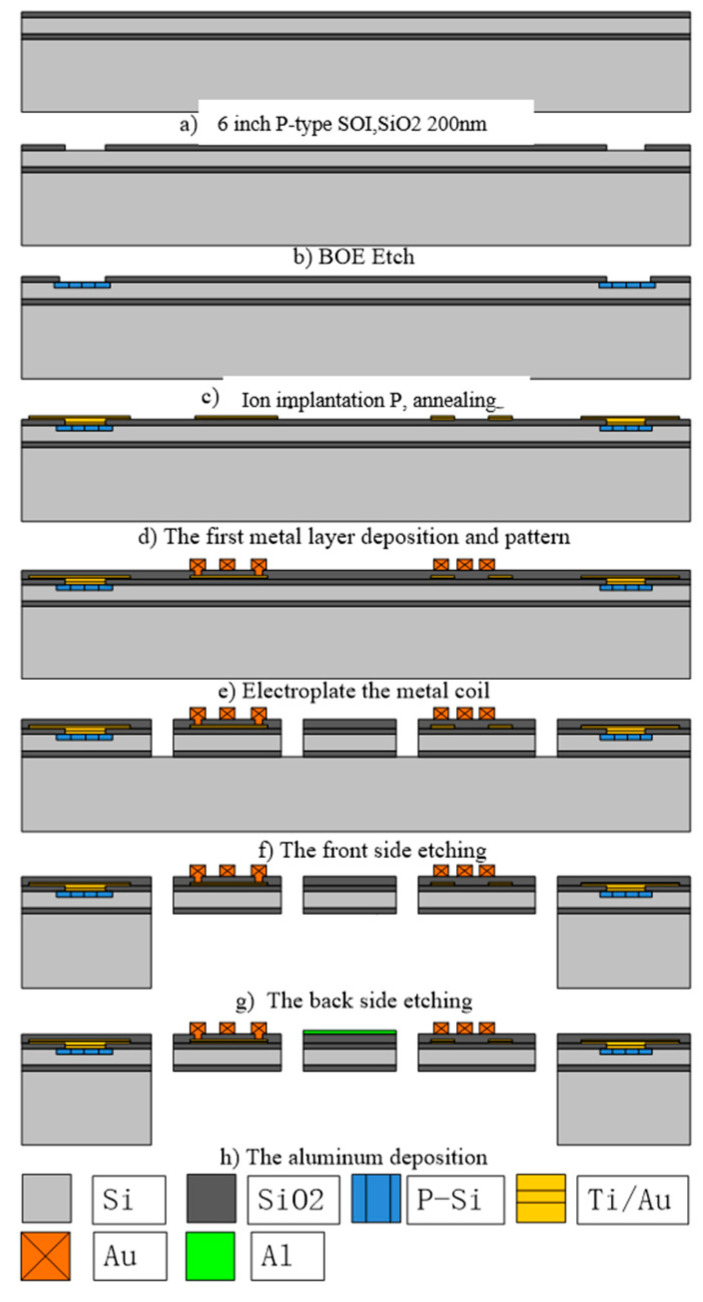
Fabrication process of the mirror.

Figure 6 shows the fabrication process of the MOEMS micromirror. The mirror structure and piezoresistive sensor are prepared from the device layer of a silicon-on-insulator (SOI) wafer. The thickness of the device Si(100) layer is 30 µm and the doping type is p-type. First, a thin layer (~200 nm thick) of silicon dioxide is grown on the silicon surface as shown in Figure 6a. Then, a photolithography step is performed to fabricate patterns of piezoresistive sensors. Phosphorus ions are implanted in two groups of dose doping for two sets of wafers. One is at 70 keV, 8 × 10^13^ atoms/cm^2^, and the other is at 70 keV, 2.2 × 10^14^ atoms/cm^2^. The simulation results show that the junction depth is about 0.782 µm when the injected ion energy is 70 keV, and the doping dose is 8 × 10^13^ atoms/cm^2^. The other junction depth is about 0.72 µm. Then, the doping concentration is calculated by dividing the doping dose by the junction depth. So, the doping dose of 8 × 10^13^ atoms/cm^2^ and 2.2 × 10^14^ atoms/cm^2^ corresponds to the doping concentration of approximately 1 × 10^18^/cm^3^ and 3 × 10^18^/cm^3^. After phosphorus doping, it is difficult to achieve ohmic contact with metal because the doping dose is small. It needs another doping at 70 keV, 5 × 10^15^ atoms/cm^2^ for ohmic contact area. Then the photoresist is removed and the wafer is cleaned before it is annealed in a furnace at 1000 °C for 30 min, as shown in Figure 6c. Figure 6d shows the bottom metallization step. The bottom metal layer (Ti/Au/Ti) with total thickness of 250 nm is deposited and patterned to form ohmic contacts with piezoresistive sensors. Silicon dioxide is then deposited on the surface as an insulation layer. Via holes for the electric access to the bottom metal layer is patterned and etched before a 200 nm metal (Ti/Au) seed layer is deposited to cover the whole wafer surface. The current carrying gold coil is patterned and electroplated with the thickness around 8 μm. The photoresist is then removed and the plating seed layer is etched away by ion beam etch (IBE), as schematically shown in Figure 6e. The last step on the front side is shown in Figure 6f, where the mechanical structures of the mirror and beams are patterned and etched by deep reactive ion etch (DRIE). The DRIE stops at the BOX surface.

**Figure 7:**
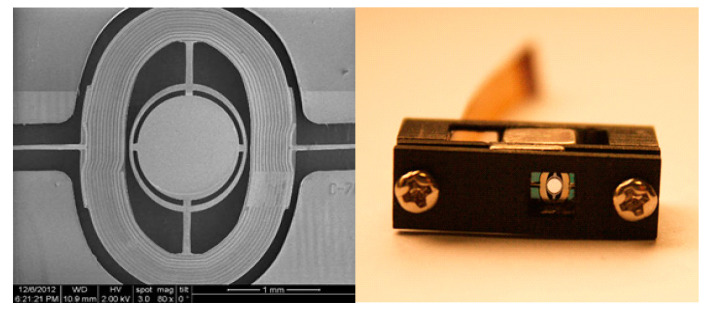
The image of the fabricated micromirror and package die assembly of the micromirror.

The wafer is then patterned on the backside for the structure releasing, as shown in Figure 6g. The handle silicon is etched away by DRIE, and the BOX layer is removed by reactive ion etch (RIE), the micromirror structures are fully released with two long beams (slow flexures) connects to the substrate. After wafer cleaning, the final step for the device fabrication is performed as shown in Figure 6h, where the metal reflective layer, usually a 100 nm thick Al, is deposited through a shadow mask in an e-beam evaporation system. Figure 7 shows the SEM picture of fabricated micromirror and the packaged micromirror with the external magnets.

## 4. Measurement Results

**Figure 8:**
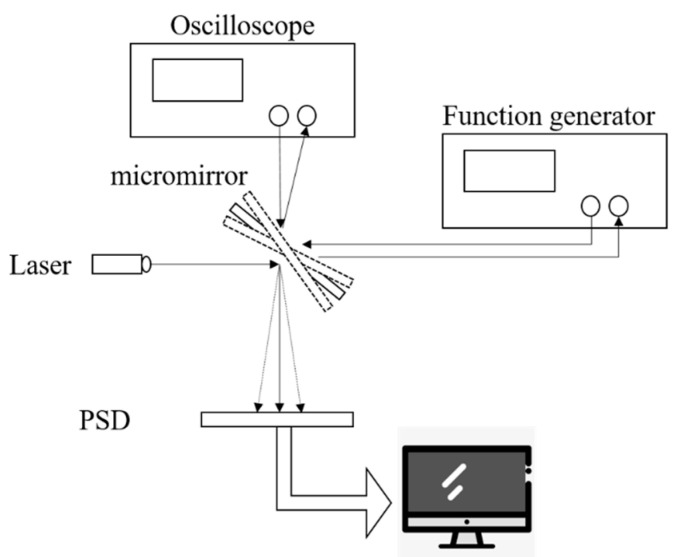
Schematic measurement setup: the 2-Dscanning mirror was driven independently by function generators [31].

**Figure 9:**
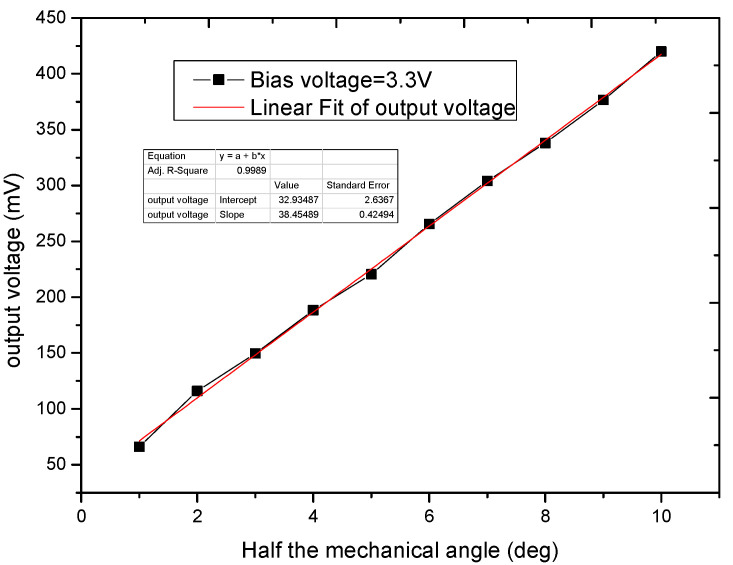
Sensor signals as the function of the mirror rotation angle of the four-terminal piezoresistive element.

Figure 8 shows the testing process of the micromirror. The function generator provides the driving signal for the micro mirror to deflect. The laser deflects from mirror surface and the light position can be precisely detected in a 10 mm × 10 mm position sensitive detector (PSD) [29,30]. The deflection angle of the micromirror can be obtained by geometric calculation. At the same time, the oscilloscope receives the output signal of the piezoresistive sensor. The supply voltage of the piezoresistive sensor is 3.3 V. The relationship between the output voltage of the piezoresistive sensor and the deflection angle obtain through the test process. Figure 9 presents the sensor signal as a function of the mirror rotation angle, and it shows very good linear relationship between the output signals and rotation angles. The input voltage for the four-terminal piezoresistive element is 3.3 V.

**Figure 10:**
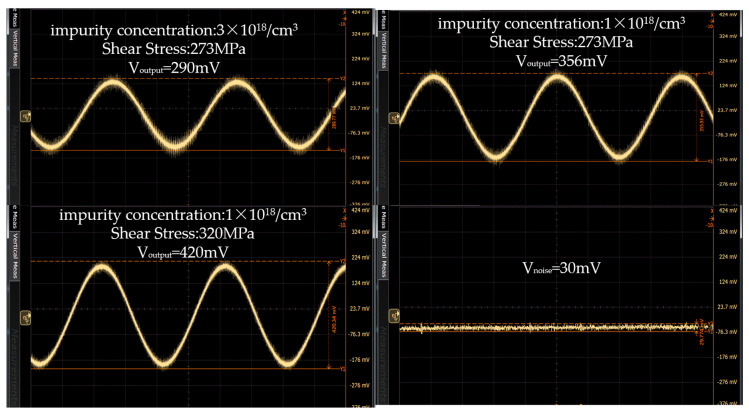
Comparison of output voltage of different impurity concentration and shear stress.

**Figure 11:**
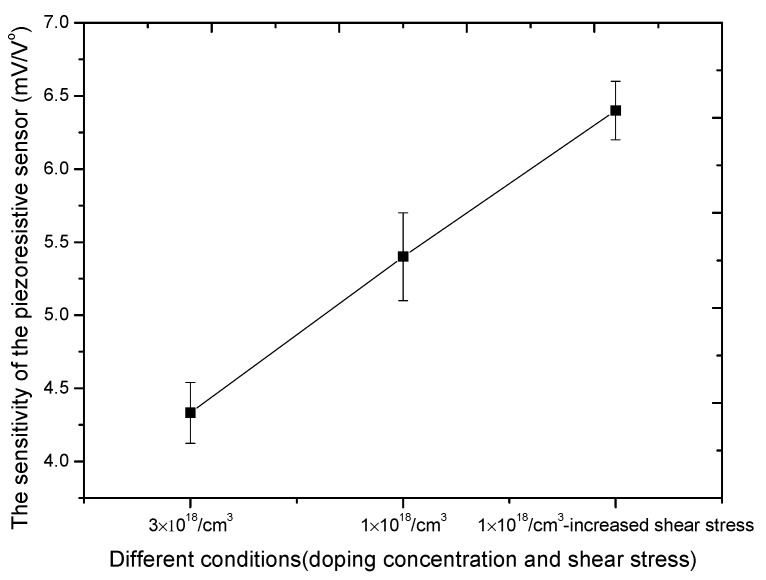
The sensitivity of the piezoresistive sensor increases with the change of piezoresistive coefficient and shear stress.

Figure 10 shows the output voltage at different doping concentrations when the deflection angle of the micromirror is stable. The supply voltage is 3.3 V and the mechanical scan angle is ±10 degrees. The piezoresistive sensor is placed in the same position on the torsional beam. When the impurity concentration is 3 × 10^18^/cm^3^, the output voltage is 290 mV. When the impurity concentration is 1 × 10^18^/cm^3^, the output voltage is 356 mV. The output voltage is increased by 20% by reducing the impurity concentration. Figure 11 shows the output voltage at different shear stresses when the deflection angle of the micromirror is stable and the impurity concentration is 1 × 10^18^/cm^3^. The output voltages are 365 and 420 mV under different shear stresses. The output voltage is also increased 15% by changing the position of the piezoresistive sensor on the torsion beam of the micromirror. As shown in Figure 10, the noise of the feedback signal is 30 mV. The SNR of piezoresistive sensor can achieve 11.4 dB. As shown in Figure 11, the increase of output voltage varies with the variation of piezoresistive coefficient and shear stress. The sensitivity of the piezoresistive sensor increased from 4.4 mV/V° to 6.4 mV/V°. The value is better than 0.42 mV/V° [26] and 5.89 mV/V° [32].

## 5. The Influence of Image Quality

In the application of laser projection, the laser beam is sent to the MEMS micromirror, and then can be reflected to the screen. Currently, the micromirror is actuated with the sinusoidal signal at 60 Hz for the slow scan, and in resonant mode for the fast scan to obtain the widest possible scanning angle at ultra-low power consumption level. The resulting raster scan motion of the laser spot on the screen is precisely controlled by the rotation of the micromirror. The colors and their intensities for every pixel on the screen are controlled directly by the modulation of RGB laser diodes. The image resolution is limited by the accuracy with which the physical position of the resonating mirror can be estimated. The position estimation is achieved by parameter estimation circuits, which include comparators, filters and peak detectors. The parameter estimation circuits provide a zero-crossing time, which is used to estimate the physical position of the resonating mirror and provide a trigger signal to excite the laser.

**Figure 12:**
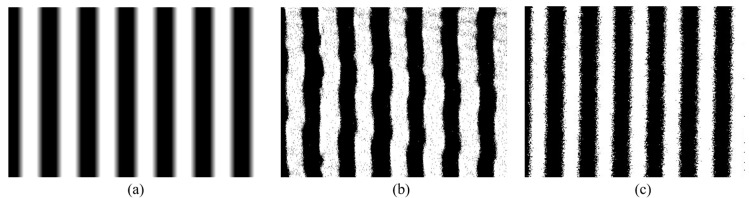
(**a**) The origin image of light and dark stripes to be displayed by the Optical-Microeletromechanical Systems (MOEMS) projection system, the image which is collected by the CCD is calculated by the binary method; (**b**) the CCD captured image projected by the micromirror with the feedback sensitivity is 4.4 mV/V°, where the pixel error is about 1.5 pixels; (**c**) the CCD captured image projected by the micromirror with the feedback sensitivity is 6.4 mV/V°, and the pixel error is about 0.5 pixels.

As shown in Figure 12a, the light and dark stripes are projected through the MOEMS laser projection module. Figure 12b,c are the projected picture. They use the same projection optical system and circuit, the only difference is the replacement of micromirror. One has a high sensitivity and the other has a low sensitivity. From Figure 12b, the edge of the projected vertical stripe has a displacement error. It is not a stable dislocation and changes over time. The error is caused by the inaccurate judgment of the zero-crossing time which is the laser starting lighting time of each line.

**Figure 13:**
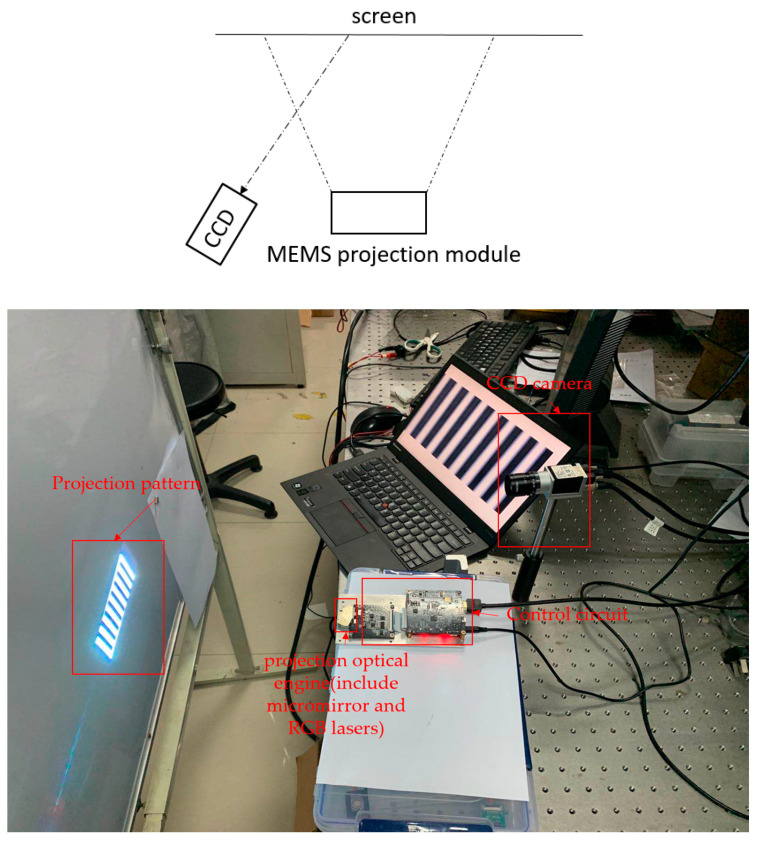
Schematic measure pixel error of the MEMS projection module.

The displacement error is not easy to measure in a single projection image. When the original picture is stable, the displacement error is also different among the many projection images. Figure 13 shows the measurement of the displacement error of the projection image. The MOEMS projection module projects vertical stripes of light and dark like Figure 12a, the bright and dark stripes are both 8 image pixels. The projection image is black and white pattern which is projected by the RGB lasers. The CCD camera collects the projection picture and by adjusting the distance between the CCD and the screen, a bright stripe has 80 CCD pixels in the image of the CCD camera. One pixel of the image projected by the module presents 10 pixels in the image of the CCD camera. The exposure time of the CCD camera sets as the refresh time of each frame of the image, for example, the image is 60Hz which is corresponding to the exposure time of 16.66ms. The whole test system includes a CCD camera and a MOEMS projection module is on an optical platform. It is removing the effect of vibration when the images are taken.

As shown in Figure 12b,c, images are collected by the CCD camera. They are images integrated with 60 photos taken with CCD in one second. Then, through binary method calculation, it can be easy to measure the maximum displacement error in one second. The pixel error of the projected image can be obtained by measuring the length of the dislocation between the line pixels. The pixel error is 1.5 pixels in Figure 12b and 0.5 pixels in Figure 12c. Figure 12b,c are projected by different micromirrors. The main difference is that the piezoresistive sensor sensitivities of the micromirrors are different. One is 4.4 mV/V° and another is 6.4 mV/V°. As the same set of optical system and control circuit are used for the micromirrors with different piezoresistive sensitivities, the test results demonstrate that the enhancement of the feedback voltage of the piezoresistive sensor can reduce the displacement error of the image. So it improves the imaging accuracy of the MOEMS projection module. However, the improvements are not strictly proportional to sensitivities of the piezoresistive sensors.

## 6. Conclusions

For this paper, a MOEMS micromirror integrated with piezoresistive sensors was fabricated, and several methods were proposed to improve the feedback voltage of the piezoresistive sensors. When the supply voltage and the mechanical scan angle were fixed, by reducing the impurity concentration from 3 × 10^18^/cm^3^ to 1 × 10^18^/cm^3^ and increasing shear stress on piezoresistive sensor, the sensitivity of the piezoresistive sensor increased from 4.4 mV/V° to 6.4 mV/V° which further contributed to an enhancement in the imaging accuracy of MEMS projection module. Specifically, the image pixel error reduced from 1.5 pixels to 0.5 pixels. The enhancement of the piezoresistive sensitivity had a positive effect on the improvement of the imaging accuracy. The results will enable us to improve the piezoresistive sensitivity in future designs.

## Figures and Tables

**Table 1:** The calculated results of the piezoresistive coefficients.

**Parameters**	**Torsional Beam along the <110> Direction**		**Torsional Beam along the <100> Direction**	
	p-Type	n-Type	p-Type	n-Type
***π*_11_(10^−11^/Pa)**	6.6	−102.2	6.6	−102.2
***π*_12_(10^−11^/Pa)**	−1.1	53.4	−1.1	53.4
***π*_44_(10^−11^/Pa)**	138.1	−13.6	138.1	−13.6
**Θ(deg)**	45	45	0	0
**Φ(deg)**	45	45	45	45
**Coefficient(10^−11^/Pa)**	7.70	−155.60	138.1	−13.6
